# Impact of the COVID-19 Pandemic on Inpatient Antibiotic and Antifungal Drug Prescribing Volumes in Germany

**DOI:** 10.3390/antibiotics13090837

**Published:** 2024-09-03

**Authors:** Winfried V. Kern, Michaela Steib-Bauert, Jürgen Baumann, Evelyn Kramme, Gesche Först, Katja de With

**Affiliations:** 1 Division of Infectious Diseases, Department of Medicine II, University Hospital and Medical Centre, and Faculty of Medicine, Albert-Ludwigs-University, 79106 Freiburg, Germany; michaela.steib-bauert@uniklinik-freiburg.de (M.S.-B.); gesche.foerst@pharmazie.uni-freiburg.de (G.F.); 2 Akademie für Infektionsmedizin, 10789 Berlin, Germany; 3 Central Pharmacy, Medius-Kliniken, 73760 Ostfildern-Ruit, Germany; j.baumann@medius-kliniken.de; 4 ADKA-Bundesverband Deutscher Krankenhausapotheker, 10559 Berlin, Germany; 5 Department of Infectious Diseases and Microbiology, University Hospital Schleswig-Holstein Campus Lübeck, University of Lübeck, 23562 Lübeck, Germany; evelyn.kramme@uksh.de; 6 DGI-Deutsche Gesellschaft für Infektiologie, 10789 Berlin, Germany; katja.dewith@ukdd.de; 7 Institute of Pharmaceutical Sciences, Faculty of Chemistry and Pharmacy, Albert-Ludwigs-University, 79085 Freiburg, Germany; 8 Institute of Infectious Diseases, University Hospital Carl Gustav Carus, Technical University, 01307 Dresden, Germany

**Keywords:** antibiotic use, hospital antibiotic consumption, coronavirus pandemic, antimicrobial stewardship, antimicrobial resistance

## Abstract

Background: Previous studies found that the coronavirus disease 2019 (COVID-19) pandemic had a variable impact on the consumption of antimicrobial drugs in human medicine, with trends in several European countries differing between community and inpatient prescribing. Aim: This study analysed changes in the volumes and use density of antibacterial and antifungal drugs dispensed in acute care hospitals in Germany between 2019 and 2022. Methods: Surveillance data for the four years available from 279 hospitals were expressed as the total volumes of daily doses or as use density (daily doses per 100 patient/occupied bed days) per year and analysed descriptively, using recommended hospital-adapted daily dose definitions (RDDs) and (as sensitivity analysis) WHO/ATC-defined daily dose definitions (DDD). Hospitals were stratified according to size (number of beds), university affiliation, and location (East, West, South). Results: There were significant decreases in both the total number of patient days and antibacterial drug volumes in 2020 through 2022 compared with 2019. The relative changes between 2019 and 2020, 2021, and 2022 were −12.8%, −13.5%, and −13.3% for patient days, and −9.7%, −11.0%, and −10.1% for antibacterial RDDs, respectively. Broad-spectrum betalactams, notably piperacillin–tazobactam and carbapenems, increased in volume, unlike most other drug classes. The resulting antibacterial drug use density was slightly but significantly increased, with pooled means (and medians) of 43.3 (40.0) RDD/100 in 2019 compared to 44.8 (41.7), 44.5 (40.80), and 44.9 (41.7) RDD/100 in the years 2020 through 2022, respectively. Antifungal drug volumes and use density increased after 2019 and peaked in 2021 (the difference between 2019 and 2021 for total volumes was +6.4%, and that for pooled mean use density values was +22.9%, respectively). These trends were similar in the different hospital strata and comparable when DDDs instead of RDDs were used. Conclusions: Similar to what has been observed in a majority of European countries, the total volume of antibacterial drug use in German acute care hospitals decreased with the pandemic, without a rebound phenomenon in 2022. In association with restricted hospital capacities and presumably more immunocompromised general medicine patients, however, inpatient prescribing of (primarily broad-spectrum) antibacterials and of antifungal drugs increased.

## 1. Introduction

The recent coronavirus disease 2019 (COVID-19) pandemic has led to major challenges in healthcare management [1,2,3]. The increased and fluctuating demand for emergency and intensive care over many months and seasons contrasted the limited staff, equipment, and infrastructure, counteracting capacity building and expansion. Consequently, surge capacities in hospitals could only be created by reducing elective care, by lowering the inflow of other emergency patients, and by structural reorganizations of wards and personnel. Substantial disruptions to primary care and public health interventions, such as lockdowns, stay-at-home orders, school closures, and travel restrictions, added further extraordinary constraints on healthcare services and on the coordination between primary and secondary care. In addition, in the early stages of the pandemic, there were a slow flow of information and much uncertainty about the resources, type of healthcare, and knowledge needed for adequate COVID-19 management. In this context, concern was expressed about the possible overtreatment, using antibiotics, of patients with confirmed or suspected COVID-19 and pulmonary infiltrates and about the risk of nosocomial transmission of potential pathogens with subsequent further increase in antimicrobial treatment [4,5,6]. The published data on these topics, however, have been conflicting and show that the impact of the pandemic on antimicrobial drug use has been highly variable.

In European countries, the monitoring of antimicrobial consumption by the ECDC has shown a consistent decrease in the community prescribing of antibiotics during the COVID-19 pandemic [7,8]. All told, 26 out of 27 EU/EEA countries (96%) reporting data for the period 2019 through 2022 observed a decreasing outpatient antibiotic use in 2020 compared with 2019, and most reported a similar decrease persisting in 2021, with a rebound in 2022. Patterns of the in-hospital prescribing of antibiotics in Europe, in contrast, have been less clear and more varying [8]. In 2020, only 17 out of 26 countries reporting hospital consumption of antibiotics (65%) showed a decrease compared with 2019. This number increased to 21 in 2021 and decreased again to 17 in 2022, with relative changes of roughly −2% and −8% in the population-weighted mean (hospital) consumption in 2020 and 2021 compared to 2019, respectively.

We were interested in assessing the pandemic-associated changes in the hospital consumption of antibacterial and antifungal drugs in German acute care hospitals. So far, these data have not been included in the ECDC annual report. Due to the large population of Germany, the data add relevant information for assessing the overall trends in population-weighted community versus inpatient antibiotic prescribing in association with the pandemic in Europe. They also provide evidence of inpatient prescribing of antifungal drugs that was dissimilar to trends in hospital antibacterial drug consumption.

## 2. Results

A total of 279 acute care hospitals were included. Table 1 shows that most of the hospitals were small-sized and more often located in Western Germany than in the East or South. They represented 18% of all general hospitals in Germany and 23% of nationally reported patient days for general hospitals. Very small hospitals were underrepresented in the sample. Seven hospitals were excluded from the analysis of antifungal drugs because of incomplete data.

### 2.1. Changes in Patient Days and Drug Volumes

**Figure 1:**
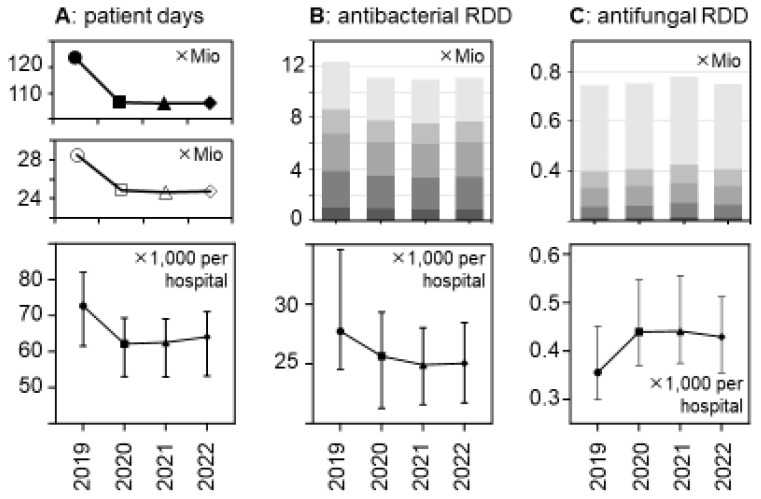
Changes between 2019 and 2022 in patient days and antibacterial and antifungal drug use volumes (in RDDs) in German acute care hospitals. (**A**) The upper panel shows the number of patient days in all German general hospitals; the middle panel shows the number of patient days in the acute care hospital sample (n = 279) of the present study, and the lower panel shows the median patient days per participant hospital (±95%CI). (**B**) The upper panel depicts the total number of antibacterial drugs (in RDDs) dispensed in the participant hospitals, stratified by hospital size/type (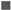, very small; 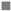, small; 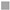, medium-sized; 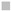, large; and 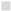, university hospitals); the lower panel shows the median antibacterial RDDs per participant hospital (±95%CI). (**C**) Similar to B for antifungal drugs (n = 272 hospitals). The changes over time were statistically significant in a hospital-level analysis (lower panels) according to a Friedman one-way repeated measure analysis of variance by rank and after Dunn’s post hoc multiple comparisons (of each year versus 2019).

There was a major reduction in hospital bed occupancy in 2020 that persisted in 2021 and 2022 and this resulted in substantial decreases in the total number of patient days, from 28.5 mio in 2019 to <25 mio in the subsequent years (Figure 1 and Supplementary Table S1). The relative decreases were similar in the different hospital strata and consistent with the national decreases. They were statistically significant in a hospital-level analysis (Figure 1), with median numbers of patient days (×1000) per hospital (±95%CI) changing from 72.68 (91.7–112.9) in 2019 to 62.16 (79.7–98.7), 62.54 (78.82–98.08), and 64.06 (79.1–98.2) in the following years 2020, 2021, and 2022, respectively (*p* < 0.0001 for each comparison).

The total volumes of dispensed antibacterial drugs (in RDDs) also decreased by roughly 10%, while the volume of antifungal drugs increased, in particular in 2021 (+6.4% versus +1.4% in 2020 and +0.4% in 2022, respectively) (Figure 1 and Table 2). These trends appeared to be consistent across the hospital size/type strata (Table 2 and Supplementary Table S1) and were statistically significant (Figure 1).

**Figure 2:**
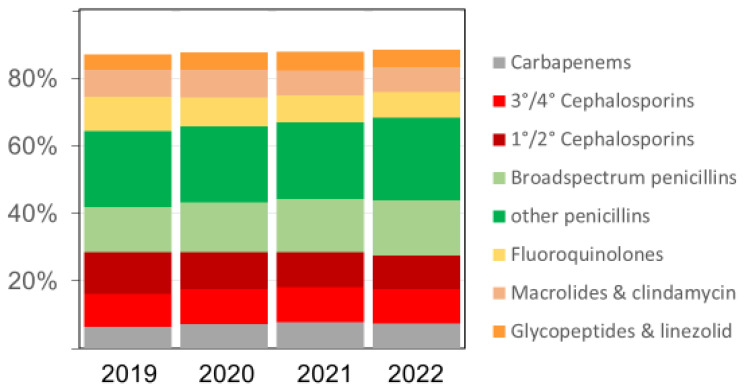
The relative proportion (%) of various antibacterial drug classes among all antibacterial drugs (in RDDs) dispensed in the acute care participant hospitals per year; 3°/4° Cephalosporins = third- and fourth-generation cephalosporins, including the new agents ceftazidime-avibactam, ceftolozane-tazobactam, ceftaroline, and cefiderocol; 1°/2° Cephalosporins = first- and second-generation cephalosporins. Broad-spectrum penicillins = piperacillin and piperacillin-tazobactam.

The overall pattern of antibacterial drugs/drug classes over time remained similar (Figure 2), but we observed several relevant changes. The proportion of carbapenems among all antibiotics, for example, increased from 6.4% in 2019 to 7.2% in 2020, 7.7% in 2021, and 7.4% in 2022, respectively. The proportion of broad-spectrum penicillin (essentially piperacillin–tazobactam) doses also increased in the same time, rising from 13.5% in 2019 to 14.7%, 15.8%, and 16.2% (in 2020, 2021, and 2022), respectively. Substantial relative (and absolute) decreases were observed for fluoroquinolones and first/second-generation cephalosporins, while there were no major changes over time in the volumes of macrolides (Figure 2 and Table 3). The increases in antifungal drug prescribing primarily affected echinocandins and azoles other than fluconazole.

### 2.2. Drug Use Density Trends

**Figure 3:**
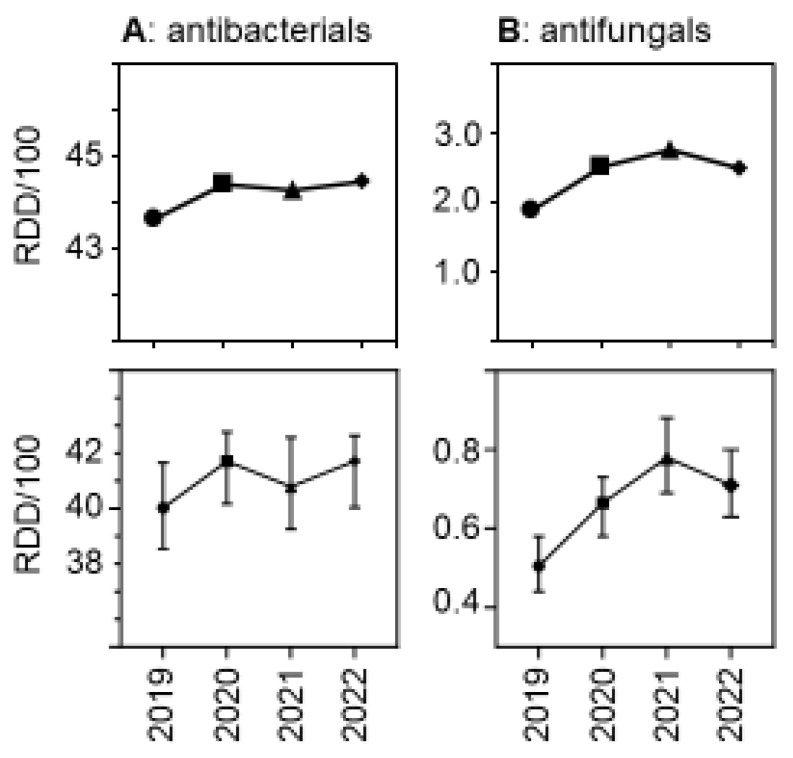
Antibacterial (**A**) and antifungal (**B**) drug use density (RDDs per 100 patient days) in the acute care participant hospitals per year. The upper panels show the pooled means and the lower panels show the medians (±95%CI). The differences were statistically significant in a hospital-level Friedman one-way repeated measure analysis of variance by ranks.

Due to the relatively greater reduction in patient days versus drug volumes, the use density (in RDDs per 100 patient days per hospital) increased. Figure 3 shows the trends over time, both for antibacterial and for antifungal drugs. The relative increase was greater for antifungal drugs than for antibacterial drugs, with a peak in antifungal use observed in 2021. As expected, drug use density was much higher in university hospitals than in the other participant hospitals, but the trends over time in the different hospital strata were similar (Supplementary Figure S1). 

We confirmed some of the changes in the use density for different antibacterial drug classes that were expected based on the changes in the drug volumes. For example, increasing use density was observed for piperacillin–tazobactam (as the main broad-spectrum penicillin) and for carbapenems, but there was no increased inpatient prescribing of macrolides and clindamycin associated with the pandemic (Supplementary Figure S2). Most of the trends appeared to be consistent across the different hospital strata (Supplementary Figure S2). The use densities for fluoroquinolones and first- and second-generation cephalosporins clearly decreased during the study period, with the latter falling most likely due to the reduced need for prophylaxis in elective surgery.

Antibacterial drug use density was consistently lower in the East than in the other regions (Supplementary Figure S1), but the number of participant hospitals from the East was low. In addition, the proportion of very small and small hospitals was higher in the East and this may account for these findings. The increase over time seemed to occur later in the East than in the other regions (Supplementary Figure S1). The antifungal drug use density per hospital in the three regions, in contrast, showed similar levels over time, with peaks in 2021 (Supplementary Figure S1).

### 2.3. Sensitivity Analyses Using Antibacterial DDD and Extrapolation to National Consumption

The results for the antibacterial drugs were similar when DDDs were used instead of RDDs (Supplementary Figure S3). However, in general and as expected [9,10], the values were higher. Total volumes decreased (Supplementary Table S1) and drug use density increased slightly (Supplementary Figure S3). These trends were significant in hospital-level analysis.

The total DDD volumes were extrapolated to the national general hospital system and population, taking into account hospital size. The resulting national estimates for total hospital consumption (Supplementary Table S2), normalised to the general population (DDD per 1000 population and day), changed from 2.07 in 2019 to 1.84 in 2020 and remained similar (1.81 and 1.80) in 2021 and 2022, respectively. Since these calculations did not include pediatric divisions, psychiatry/psychosomatics hospital services and other non-general (monospecialty) hospitals, the true values are likely to be somewhat higher.

## 3. Discussion

The major finding of this study was that the total volumes of antibacterial drugs, but not of antifungal drugs, decreased with the reduced hospital bed occupancy in 2020 and thereafter in association with pandemic changes. The overall decrease of roughly 10% in hospital consumption of antibacterial drugs is relevant, noteworthy, and probably representative of the German hospital system. In our opinion, the decrease was primarily driven by the constraints on healthcare services during the pandemic and the associated structural changes seen with the reorganization of wards, less elective care, more intensive care, and the increased emergency admission of elderly patients with complex, and probably more, advanced diseases [11,12,13,14,15]. 

Reduced hospital consumption of antibiotics, associated with the pandemic, has been observed in a majority of but not all European countries, as reported by the ECDC [8]. Interestingly, the reduced consumption appeared to be enhanced in 2021 and attenuated in 2022, a pattern that we did not observe in Germany. There is limited information from European countries about the patterns of drug use density per admission or per patient days during the pandemic. Some of the available in depth-analyses with use density data only cover the first or first two years of the pandemic. In Italy and France, for example, no increased use density was observed in 2020 compared with 2019 [16,17]. Switzerland reported an overall decrease in hospital antibiotic consumption in 2020 compared with 2019, and a simultaneous (small) increase in antibiotic use density that primarily included broad-spectrum antibiotics [18,19]. Conversely, studies from Hungary and Croatia reported a massive increase in hospital antibiotic use density in 2020, with no decrease in overall consumption [20,21]. Similar to the findings in the present study, Denmark reported a large increase in antibiotic use density after 2019 that persisted (at least) until 2022, while the total consumption decreased [22]. Decreasing overall antibiotic consumption in hospitals in 2020 and 2021 compared with 2019 was also seen in Sweden and the Netherlands [23,24]. In Dutch hospitals, the inpatient antibiotic use density increased at the same time between 2019 and 2020. Together with reports from other European and non-European countries [25,26,27,28,29,30], these investigations show varying trends, although reports of increased hospital antibiotic use density prevail. An important factor in the variability was likely the differential pandemic dynamics across (European) countries and regions and the varying type and timing of public health interventions, structural changes in the hospital systems, and primary care in response to these dynamics. It will be interesting to evaluate longer-term trends in those countries for which the reports focussed only on the immediate changes associated with the first or first and second pandemic surges. 

A second important finding of this study was the impact on broad-spectrum antibiotic prescribing, notably of carbapenems and piperacillin-tazobactam, and on antifungal prescribing, which increased while most other drug classes (with the exception of glycopeptides/linezolid) decreased in total dispensed volumes and some decreased even in use density. Such a pandemic-associated shift towards broad-spectrum betalactams has been described by other investigators [6,16,17,18,19,20,21,22,23,24,31,32,33,34,35], but less is known about the trends in the hospital prescribing of antifungals. Hospital-onset invasive *Candida* infection and, more rarely, mould infections have been associated with severe COVID-19 cases. Epidemiological studies documented increased incidences following pandemic waves [36,37,38,39,40,41,42]. Immunosuppressants are predisposing factors in this context, and these were recommended as adjunctive therapies in severe COVID-19 patients as early as 2020 (dexamethasone) and 2021 (anti-IL-6) [43,44,45,46]. There has been some concern about the role of early invasive versus non-invasive ventilation and the frequent extracorporeal membrane oxygenation (ECMO) therapies that have likely increased the risk for superinfection. A predisposing factor for fungal superinfection in this context may also have been the increased prescribing of broad-spectrum antibiotics. Both broad-spectrum antibiotics and antifungal agents may have been (too) often empirically prescribed because of clinical and diagnostic uncertainties, in particular in long-stay intensive care patients [47,48]. 

Few studies have examined the impact these developments had on antifungal drug consumption. In Dutch and Spanish hospitals, the antifungal use density increased by 10–12% in 2020 compared to 2019 [24,25]. Four French health centres observed an increase in voriconazole consumption in 2020 compared with 2019, and this rise was particularly large in intensive care [49]. In a study from the United Kingdom, there was no change in inpatient antifungal prescribing [50]. In the most recent ECDC report [8], the total consumption (combining community and hospital sectors if data available) of systemic antifungals (excluding terbinafin) in the population decreased in 2020 in a majority of reporting countries, but this finding is difficult to interpret since hospital prescribing could not specifically be evaluated. We are not aware of other multicenter studies examining the longer-term trends of inpatient antifungal prescribing covering pandemic-associated changes.

The strength of the present study is the relatively large number of hospitals with complete data over the four years of study, with the option to perform stratified analysis according to hospital size and repeated measures analyses. Another strength is the provision of reliable antifungal consumption data, which are missing in many reports. Potential limitation include the annual (instead of quarterly) data, which did not capture the shorter-term dynamic trends associated with pandemic waves, and responses, which may vary even in the different regions of the same country. Another limitation is that the (pre-pandemic) baseline period covered only one year, not taking into account the previous variations in hospital drug use over time. Finally, the metrics we used—RDDs and DDDs instead of days of therapy, normalisation with patient days versus with admissions—have their inherent limitations that need to be considered when interpreting and comparing the results. 

In summary, we show that in one of the largest EU countries, the total volume of antibacterial drugs prescribed in acute care hospitals substantially decreased with the pandemic without a rebound phenomenon in 2022, which is relevant at the population level. As shown by use density data, inpatient prescribing, particularly of broad-spectrum antibiotics but also of systemic antifungal agents, however, increased, presumably in relation to the different case mix in the pandemic situation of more vulnerable and critically ill patients. 

## 4. Materials and Methods

### 4.1. Setting and Definitions

We used data collected by the so-called ADKA-if-DGI surveillance programme (https://www.antiinfektiva-surveillance.de, accessed on 30 May 2024), which receives data on dispensed antimicrobial drugs from hospital pharmacies and uses patient days (occupied bed days) as a denominator. The data comprise all drugs of the ATC groups J01, J02, and J04AB02 (rifampicin, if not given as fixed combination), dispensed to all inpatient divisions of a given hospital except to psychiatry/psychosomatics and pediatrics. The program stratifies participant hospitals according to size: very small hospitals (<200 beds), small hospitals (200–399 beds), medium-sized hospitals (400–800 beds) and large hospitals (>800 beds); among the large hospitals, university hospitals were evaluated separately.

The drug use volumes are converted into hospital-adapted “recommended” daily doses (RDDs) and WHO-ATC-defined daily doses (DDD, 2023 version) and usually expressed as RDDs (DDDs) per 100 patient days (RDD/100 or DDD/100). Comprehensive quarterly reports are compiled and made available to the antimicrobial stewardship teams of each participant hospital for feedback purposes. Acute care hospitals, participating continuously in this (non-compulsory) programme in the years 2019 through 2022 and reporting complete data, were included in the present analysis.

### 4.2. Analysis and Statistics

We calculated total drug volumes (in daily doses) and the pooled means and medians (with 95% confidence intervals) per hospital and per 100 patient days. Comparisons of absolute counts between the years are reported as relative differences (in percentages). The statistical significance of changes over time was assessed by the non-parametric Friedman test for repeated paired-group measures and Dunn’s multiple pairwise comparison (2019 versus each of the following years) with a Bonferroni adjustment (used as a post hoc test). Statistical tests were two-tailed and considered significant if the *p* value was <0.05; this was calculated with GraphPad Prism V.6 (GraphPad Software, La Jolla, CA, USA). Additional exploration of the data included an assessment of the influence of hospital location (East, West, South) and hospital size strata.

For the comparison with national data and an extrapolation of the DDD results, we used the annual data published by the Federal Office of Statistics (https://www.destatis.de, accessed on 30 May 2024) for general hospitals (with the corresponding hospital size/type strata) and the total population. For each hospital size stratum, we obtained the national data on patient days for each year of study and calculated the extrapolated national DDDs with the formula: study DDD/study patient days × national patient days. We then summed up the extrapolated national DDDs of each stratum to give the overall national DDDs and used the numbers for the national population of each year to estimate the (extrapolated) number of DDDs per 1000 inhabitants and day, which is the metric used by the ECDC.

## Figures and Tables

**Table 1:** Number of participant hospitals in the different strata.

Hospital Size/Type	Total	Location *		
		East	West	South
Non-university hospitals				
<200 beds	83	20	24	39
200–399 beds	103	25	44	34
400–800 beds	54	6	32	16
>800 beds	17	5	7	5
University hospitals	22	4	10	8
Total	279	60	117	102

* The different regions (hospital location) were defined as East (federal states of Berlin, Brandenburg, Mecklenburg-Vorpommern, Sachsen, Sachsen-Anhalt, and Thüringen), West (Bremen, Hamburg, Hessen, Niedersachsen, Nordrhein-Westfalen, Rheinland-Pfalz, Saarland, and Schleswig-Holstein), and South (Bayern, and Baden-Württemberg).

**Table 2:** Relative changes (%) in overall antibacterial and antifungal drug volumes (RDDs, pooled data) (index year 2019).

Hospital Size/Type	Antibacterial Drugs (% Change)			Antifungal Drugs (% Change)		
	2020	2021	2022	2020	2021	2022
Non-university hospitals						
<200 beds	−12.5	−14.1	−13.2	+18.7	+36.0	+3.0
200–399 beds	−8.1	−12.9	−11.1	+6.9	+22.1	+12.5
400–800 beds	−9.2	−10.9	−8.1	−0.9	+2.4	+1.6
>800 beds	−10.3	−12.1	−11.6	+4.9	+12.3	−0.2
University hospitals	−8.6	−8.2	−9.2	−0.4	+3.0	−0.9
Total (% change)	−9.7	−11.0	−10.1	+1.4	+6.4	+0.7

**Table 3:** Relative changes (%) in antibacterial drug consumption (RDDs, pooled data) (index year 2019) by different drugs/drug classes.

Antibacterial Drug/Class	% Change		
	2020	2021	2022
Carbapenems	+1.2	+7.0	+4.0
3°/4° Cephalosporins	−4.6	−5.3	−6.4
1°/2° Cephalosporins	−19.2	−25.1	−26.7
Broad-spectrum penicillins	−1.3	+4.5	+8.3
Aminopenicillin-BLI	−8.6	−9.8	−0.2
Narrow-spectrum penicillins	−11.6	−10.5	−5.5
Fluoroquinolones	−22.7	−30.6	−32.8
Macrolides & clindamycin	−9.5	−17.3	−18.4
Glycopeptides & linezolid	+0.4	+0.7	+1.8
others	−12.8	−15.8	−19.4
Total (pooled)	−9.7	−11.0	−10.1

Here, 3°/4° Cephalosporins = third- and fourth-generation cephalosporins, including the new agents ceftazidime-avibactam, ceftolozane-tazobactam, ceftaroline, and cefiderocol; 1°/2° Cephalosporins = first- and second-generation cephalosporins. Broad-spectrum penicillins = piperacillin and piperacillin-tazobactam. BLI = betalactamase inhibitor.

## Data Availability

The data underlying this article will be shared in aggregated form on reasonable request to the corresponding author.

## Supplementary Materials

The following supporting information can be downloaded at: https://www.mdpi.com/article/10.3390/antibiotics13090837/s1, Table S1: The number of patient days and of RDD and DDD (antibacterial drugs) in the participant hospitals by year, and rela-tive changes of patient day numbers (%) compared with the index year 2019; Figure S1: Antibacterial and antifungal drug use density (RDD per 100 patient days) in the acute care participant hospitals per year stratified by size/type of the hospital and according to location; Figure S2: Use density (RDD per 100 patient days) for different antibacterial drugs/drug classes in the acute care participant hospitals per year stratified by size/type of the hospital; Figure S3: Antibacterial drug use volumes (DDD) and density (DDD per 100 patient days) in the acute care hospital sample (n = 279) of the present study; Table S2: Extrapolated antibacterial drug DDD volumes to hospital consumption in the general population.
