# Supplementary material for: Impact of the COVID-19 Pandemic on Inpatient Antibiotic and Antifungal Drug Prescribing Volumes in Germany
Source: Antibiotics (Basel). 2024 Sep 3;13(9):837. doi: 10.3390/antibiotics13090837 (PMC11429143; doi:10.3390/antibiotics13090837)
Supplement: Supplementary file 1 [file antibiotics-13-00837-s001.zip › antibiotics-3170126-supplementary.pdf]

Supplementary tables and figures

Suppl. table S1. The number of patient days and of RDD and DDD (antibacterial drugs) in the participant hospitals by year, and relative changes of patient day numbers (%) compared with the index year 2019.

Hospital size/type	2019<br>RDD<br>DDD	patient days	2020<br>RDD<br>DDD	patient days<br>(% change)	2021<br>RDD<br>DDD	patient days<br>(% change)	2022<br>RDD<br>DDD	patient days<br>(% change)
Non-university hospitals								
<200 beds	1,006,333<br>1,229,257	2,844,048	880,403<br>1,068,614	2,416,282 (-15.0%)	864,470<br>1,054,702	2,385,777 (-16.1%)	873,850<br>1,061,787	2,375,092 (-16.5%)
200-399 beds	2,852,773<br>3,518,213	7,331,598	2,563,806<br>3,135,297	6,313,235 (-13.9%)	2,486,162<br>3,031,749	6,175,016 (-15.8%)	2,536,073<br>3,073,054	6,256,817 (-14.7%)
400-800 beds	2,883,808<br>3,540,587	6,959,943	2,618,615<br>3,193,758	6,118,385 (-12.1%	2,569,230<br>3,110,409	6,041,037 (13.2%)	2,648,921<br>3,206,537	6,142,032 (-11.8%)
>800 beds	1,865,923<br>2,268,991	4,409,049	1,672,805<br>2,008,233	3,787,703 (-14.1%)	1,639,857<br>1,950,263	3,714,227 (-15.8%)	1,649,498<br>1,958,578	3,719,380 (-15.6%)
University hospitals	3,735,752<br>4,588,976	6,994,536	3,414,574<br>4,154,706	6,255,745 (-10.6%)	3,429,743<br>4,181,605	6,361,853 (-9.0%)	3,390,900<br>4,138,263	6,237,952 (-10.8%)
Total (pooled)		28,539,174		24,891,350 (-12.8%)		24,677,910 (-13.5%)		24,731,273 (-13.3%)

Suppl. table S2. Extrapolated antibacterial drug DDD volumes to hospital consumption in the general population (*based on national statistics for pooled patient day data in the different hospital strata and for the total population [www.destatis.de]).

Hospital size/type	2019		2020		2021		2022	
	this study DDD / patient days	extrapolated DDD / patient days*	this study DDD / patient days	extrapolated DDD / patient days*	this study DDD / patient days	extrapolated DDD / patient days*	this study DDD / patient days	extrapolated DDD / patient days*
Non-university hospitals								
<200 beds	1,229,257	7,702,932	1,068,614	6,556,427	1,054,702	6,428,766	1,061,787	6,512,567
	2,844,048	17,821,747*	2,416,282	14,824,976*	2,385,777	14,542,118*	2,375,092	14,567,843*
200-399 beds	3,518,213	13,552,803	3,135,297	12,070,666	3,031,749	12,065,501	3,073,054	12,292,356
	7.331,598	28,242,662*	6,313,235	24,305,497*	6,175,016	24,574,811*	6,256,817	25,027,552*
400-800 beds	3,540,587	22,572,647	3,193,758	19,836,225	3,110,409	19,685,227	3,206,537	20,179,659
	6.959,943	44,372,398*	6,118,385	38,000,895*	6,041,037	38,232,651*	6,142,032	38,653,572*
>800 beds	2,268,991	10,027,865	2,008,233	8,786,693	1,950,263	8,308,640	1,958,578	8,160,215
	4,409,049	19,485,907*	3,787,703	16,572,472*	3,714,227	15,823,596*	3,719,380	15,496,416*
University hospitals	4,588,976	9,100,796	4,154,706	8,556,233	4,181,605	8,414,228	4,138,263	8,281,484
	6,994,536	13,871,470*	6,255,745	12,883,129*	6,361,853	12,801,325*	6,237,952	12,483,378*
(extrapolated) total DDD	62,957,043		55,806,244		54,902,362		55,426,281	
Population*	83,166,711		83,155,031		83,237,124		84,358,845	
(extrapolated) DDD per 1,000 population and day	2.07		1.84		1.81		1.80	

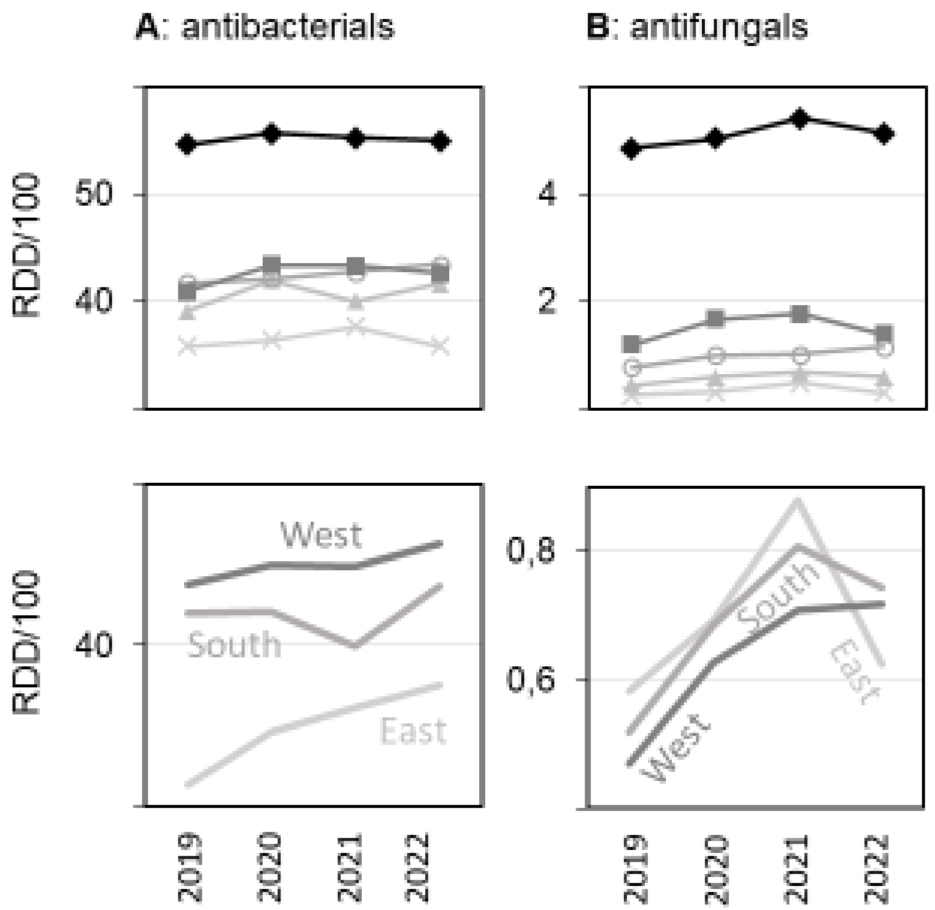

Suppl. figure S1. Antibacterial and antifungal drug use density (RDD per 100 patient days) in the acute care participant hospitals per year stratified by size/type of the hospital and according to location. The upper panels show the median values for university hospitals (◆) and for non-university large (■), medium-sized (○), small (▲), and very small (×) hospitals. The lower panels show the median values for the different regions.

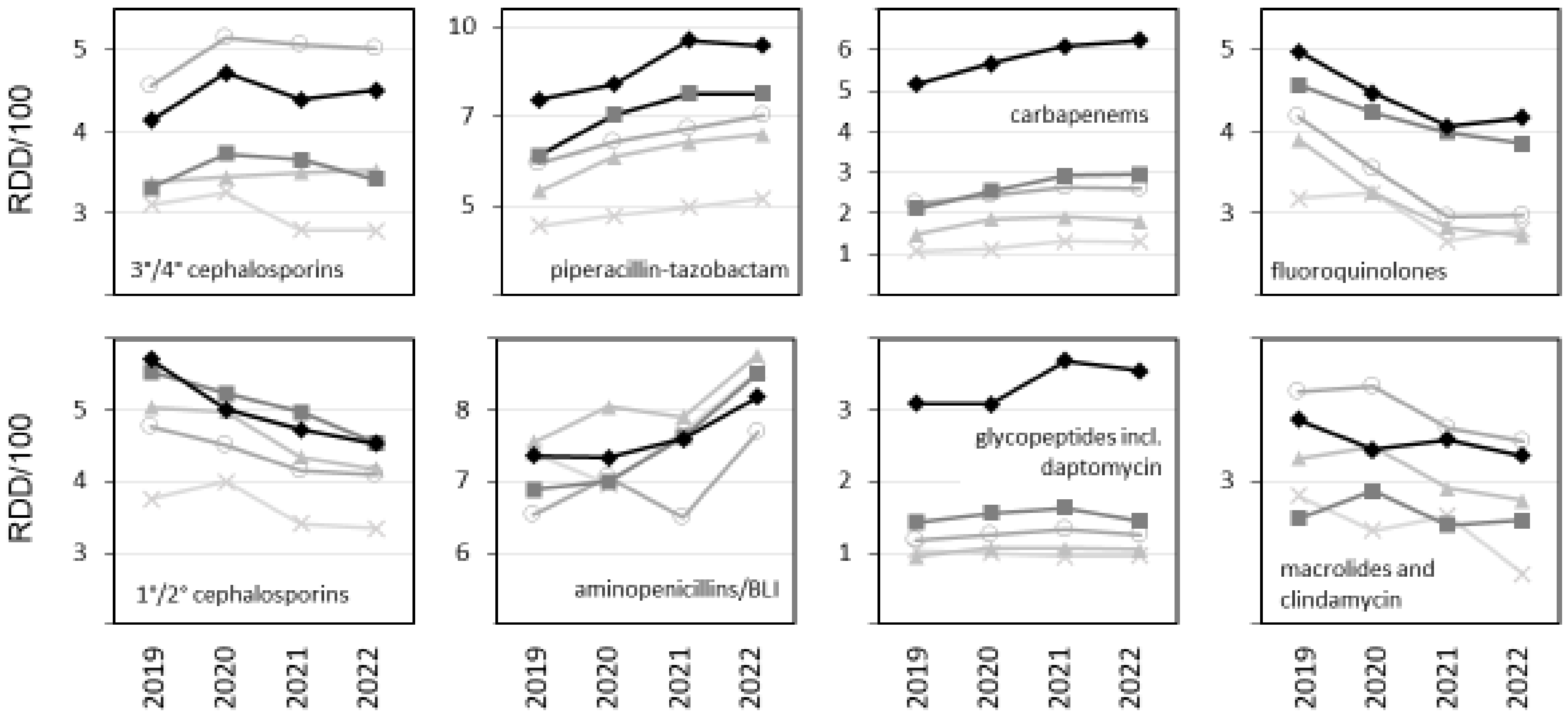

Suppl. figure S2. Use density (RDD per 100 patient days) for different antibacterial drugs/drug classes in the acute care participant hospitals per year stratified by size/type of the hospital. Shown are the median values for university hospitals (◆) and for non-university large (■), medium-sized (○), small (▲), and very small (×) hospitals.

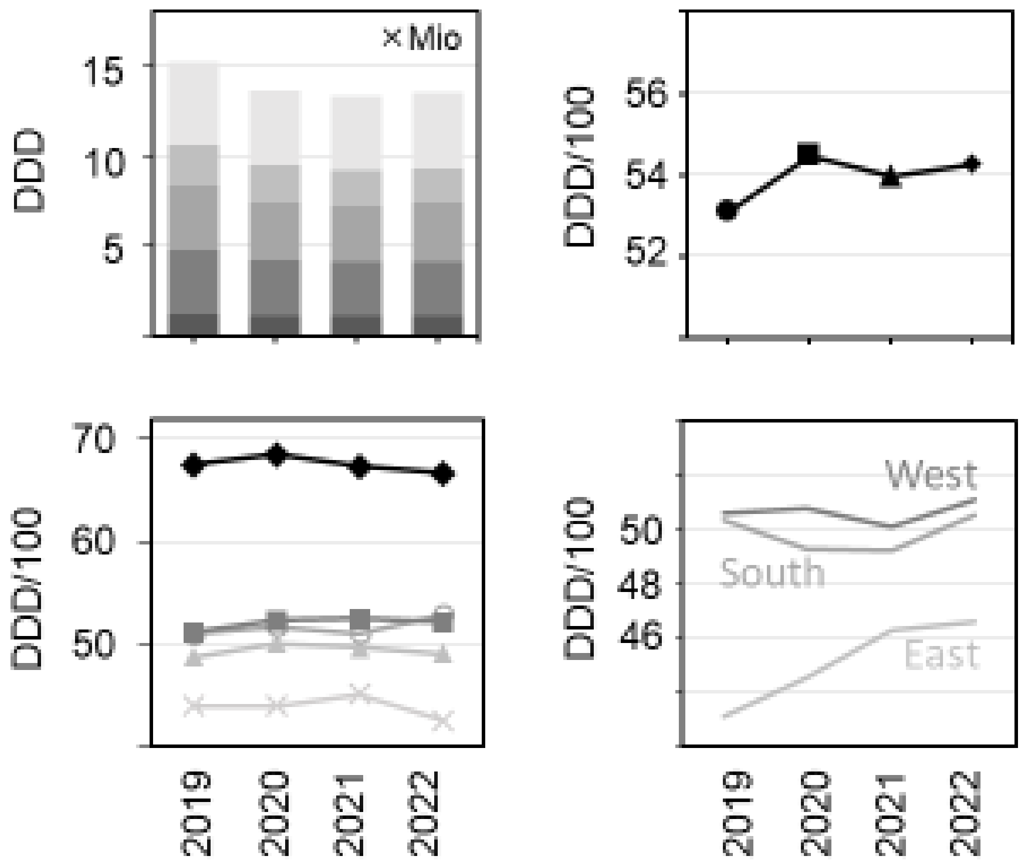

Suppl. figure S3. Antibacterial drug use volumes (DDD) and density (DDD per 100 patient days) in the acute care hospital sample (n=279) of the present study. The upper left depicts the total number of antibacterial drugs (DDD) dispensed in the participant hospitals stratified by hospital size/type (■ very small, ■ small, ■ medium-sized, ■ large, ■ university hospitals). The upper right panel shows the pooled means, the lower left panel shows median values for university hospitals (◆) and for non-university large (■), medium-sized (○), small (▲), and very small (×) hospitals, and the lower right panel show the median values for the different regions.
